# A systematic analysis of the effects of increasing degrees of serum immunodepletion in terms of depth of coverage and other key aspects in top-down and bottom-up proteomic analyses

**DOI:** 10.1002/pmic.201100005

**Published:** 2011-06

**Authors:** Matthew P Welberry Smith, Steven L Wood, Alexandre Zougman, Jenny T C Ho, Jianhe Peng, David Jackson, David A Cairns, Andrew J P Lewington, Peter J Selby, Rosamonde E Banks

**Affiliations:** 1 Clinical and Biomedical Proteomics Group, Cancer Research UK Centre, Leeds Institute of Molecular Medicine, St. James's University HospitalLeeds, UK; 2 Thermo Fisher ScientificHemel Hempstead, UK

**Keywords:** 2-D DIGE, Biomedicine, Immunodepletion, LC-MS/MS, Reproducibility, Serum

## Abstract

Immunodepletion of clinical fluids to overcome the dominance by a few very abundant proteins has been explored but studies are few, commonly examining only limited aspects with one analytical platform. We have systematically compared immunodepletion of 6, 14, or 20 proteins using serum from renal transplant patients, analysing reproducibility, depth of coverage, efficiency, and specificity using 2-D DIGE (‘top-down’) and LC-MS/MS (‘bottom-up’). A progressive increase in protein number (≥2 unique peptides) was found from 159 in unfractionated serum to 301 following 20 protein depletion using a relatively high-throughput 1-D-LC-MS/MS approach, including known biomarkers and moderate–lower abundance proteins such as NGAL and cytokine/growth factor receptors. On the contrary, readout by 2-D DIGE demonstrated good reproducibility of immunodepletion, but additional proteins seen tended to be isoforms of existing proteins. Depletion of 14 or 20 proteins followed by LC-MS/MS showed excellent reproducibility of proteins detected and a significant overlap between columns. Using label-free analysis, greater run-to-run variability was seen with the Prot20 column compared with the MARS14 column (median %CVs of 30.9 versus 18.2%, respectively) and a corresponding wider precision profile for the Prot20. These results illustrate the potential of immunodepletion followed by 1-D nano-LC-LTQ Orbitrap Velos analysis in a moderate through-put biomarker discovery process.

## 1 Introduction

Biomarkers are increasingly important in the drive to individualise healthcare. Remarkable strides in proteomic technologies have been made but the results for clinical fluid-based discovery studies are disappointing with few emerging novel markers with strong enough clinical performance to suggest that they will contribute to changing clinical practice. The existence of such powerful markers remains a real possibility but they may occur in relatively low abundance. Hence, efforts to study the lower abundance plasma and serum proteome in comparative analyses are central to progress if such studies are to realise their potential. Serum and plasma represent attractive sources of biomarkers as they are minimally invasive and potentially contain proteins and peptides shed directly by diseased tissues as well as those reflecting systemic effects of the disease process. However, using serum/plasma for biomarker discovery studies is challenging due to the wide dynamic range of protein abundances (over ten orders of magnitude) and the fact that 22 proteins make up around 99% of the total protein mass [1].

To overcome these various issues, fractionation approaches have been employed. Extensive fractionation combining immunoaffinity subtraction of the most abundant proteins with subsequent chemical fractionation based on cysteinyl peptide and *N*-glycopeptide selective captures, and analysis with 2-D LC-tandem mass spectrometry (LC-MS/MS), resulted in the identification of 1494 proteins in a trauma patient plasma sample including 78 cytokines/cytokine receptors [2]. Similarly, targeting glycosylated serum proteins using hydrazide chemistry with stable isotope labelling of glycopeptides and MS/MS [3] has enabled the identification of lower abundance proteins including carboxypeptidase N and interferon (α and β) receptor 2 [4]. However, such extensive and time-consuming fractionation is not easily compatible with biomarker discovery experiments where comparative quantitative analysis of multiple samples is required. A relatively simple approach which has been used either singly or as the first step in these more complex fractionation series is the removal of the most abundant proteins by immunodepletion [5]. However, little systematic investigation has been carried out in terms of the relative benefits of removing different numbers of abundant proteins in terms of the depth of coverage achieved in relatively rapid processing, the specificity, reproducibility, and the ease of use/compatibility of the approaches with subsequent proteomic analyses. A range of commercially produced LC columns are now available for immunodepletion including the Multiple Affinity Removal System (MARS) series of columns variably removing 6, 7, or 14 abundant proteins (Agilent, CA, USA) and the ProteoPrep20 which removes 20 proteins (Sigma-Aldrich, UK).

The MARS6 column has been most extensively used in comparative studies [6–9]. Good run-to-run variability and higher total numbers of peptide/protein identifications have been achieved, e.g. 138 protein identifications with at least two peptides for MARS6-depleted serum/plasma compared with <100 for six other strategies employing various biochemical enrichment methods when assessed by LC-MS/MS or similar approaches [6, 8]. A recent assessment of depletion with either MARS14 or MARS6 in healthy volunteer plasma samples concluded a similar 25% increase in identification was seen with either column, with 23 moderate- to low-abundance proteins detected in depleted samples (e.g. intercellular adhesion molecule 1, macrophage CSF receptor 1) [10]. Reported disadvantages of immunodepletion columns include variable depletion efficiency [8, 11] between proteins and concomitant loss of non-targeted proteins [10, 12]. Examination of the effects of removing 20 proteins is limited, but a 2-D PAGE-based study including MARS6, Seppro MIXED12-LC20, and ProteoPrep20 spin columns revealed >1200 gel features with 6 or 12 protein depletion though only 1024 with 20 depletion which was only a minimal improvement from depletion of just albumin and IgG [13]. A limited analysis of the removal of 1, 6, 12, or 20 proteins with MARS6, IgY-12 high-capacity spin column (Beckman Coulter, High Wycombe, UK) and ProteoPrep20 spin column [14] showed some additional depth of coverage, such as the detection of factor H complement protein only after depletion of 12 or 20 proteins.

We report here the results of a systematic analysis of the effects of removing either 6, 14, or 20 proteins compared with whole serum alone, comparing depth of coverage, reproducibility, ease of use, and specificity. This was achieved using pooled serum samples from patients post-renal transplantation which are likely to contain a wider range of proteins than healthy control samples, which have been often used by other studies [10, 13]. Importantly, both 2-D DIGE (top-down) and parallel LC-MS/MS analysis (bottom-up) were used as downstream readouts. The results of this study provide important insights into the use of immunodepletion in relatively simple serum-based biomarker discovery pipelines.

## 2 Materials and methods

### 2.1 Materials

MARS6 Hu6HC 4.6×100 mm column, MARS14 Hu14 4.6×100 mm column, and MARS buffers A and B were obtained from Agilent (West Lothian, UK). The Prot20 depletion column and the ProteoSilver kit were obtained from Sigma-Aldrich and 15 mL capacity, 10 kDa molecular weight cut-off (MWCO) spin-filter units were obtained from Millipore (Watford, UK). Corning Spin-X centrifuge tubes, 0.2 μm pore size were obtained from Corning (Amsterdam, The Netherlands). Bradford reagent was obtained from Bio-Rad (Herts, UK). ZEBA-spin desalt columns, 2 mL size, were obtained from Pierce (Northumberland, UK). Cy-fluorescent dyes, IPG-strips, and bovine serum albumin standard were obtained from GE-Healthcare (Bucks, UK). Sequencing-grade trpysin was obtained from Promega (Southampton, UK). All other chemicals were obtained from Sigma-Aldrich and were of analytical grade or above.

### 2.2 Samples

**Figure 1:**
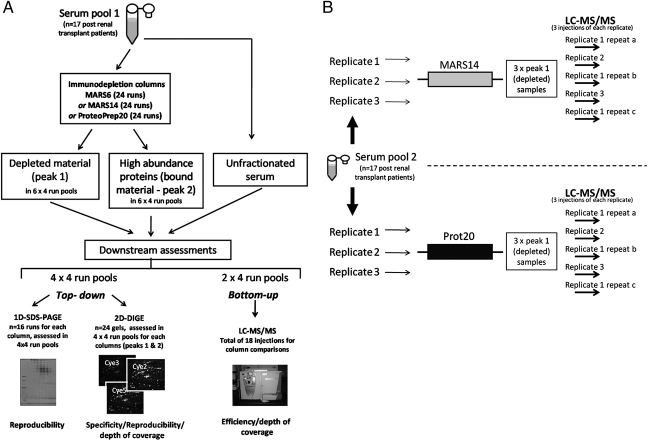
Schematic overview of the experimental approach. (A) Pooled serum (from 17 renal transplant patients) either unfractionated or following depletion of 6, 14, or 20 proteins was compared using 1-D PAGE, 2-D DIGE, and LC-MS/MS to assess reproducibility, efficiency, specificity, and depth of coverage; (B) Design of the subsequent more detailed analysis of the reproducibility of the depletion process (14 or 20 proteins) combined with LC-MS/MS. Repeat analysis of one sample replicate in each case was used to provide an indication of the reproducibility of the LC-MS/MS analysis. In each case, triplicate injections were carried out for all replicates.

A pooled serum sample was prepared by combining serum samples from 17 patients (ten male and seven female; age range, 16–68 years) at days 1–3 post-renal transplant. All samples had been obtained with informed consent and ethics approval. In each case, venous blood samples had been collected into 9 mL Z-serum clot activator tubes (Greiner Bio-One, UK), left to clot for minimum 45 min before being centrifuged at 2000×*g* for 10 min at room temperature. Serum was aliquotted and then stored at −80°C until use. The protein concentration of the starting pooled serum sample and all bound/unbound fractions after later immunodepletion were determined using the modified Bradford method (Bio-Rad) using a bovine serum albumin standard (GE Healthcare). An overview of the study elements is shown in Fig. 1.

### 2.3 Depletion of high-abundance proteins

The intended depletions of the three tested columns are albumin, IgG, IgA, transferrin, fibrinogen, α-1-antitrypsin, and haptoglobin (all columns) with additionally IgM, α-2-macroglobulin, α-1-acid glycoprotein, apolipoprotein A-I, apolipoprotein A-II, complement C3 and transthyretin (MARS14 and Prot20), and IgD, ceruloplasmin, apolipoprotein B, complement C1q, complement C4, and plasminogen (Prot20). The MARS columns were run on an Agilent 1200 series HPLC, with UV absorbance detector set at 214 nm, with proprietary buffers. Serum was filtered through 0.22 μm Spin-X filters and diluted at 1:4 with buffer A before injection (320 μL diluted serum for MARS6 and 160 μL for MARS14). Running conditions were as follows: MARS6 – max. pressure, 120 MPa, 100% buffer A at 0.5 mL/min, 0–13 min, 100%, buffer B at 1.0 mL/min, 13–20 min, 100% buffer A at 1.0 mL/min, 20–30 min; MARS14 – max. pressure 60 MPa, 100% buffer A at 0.125 mL/min, 0–21 min, 100% buffer A at 1.0 mL/min, 21–23 min, 100% buffer B at 1.0 mL/min, 23–30 min, 100% buffer A at 1.0 mL/min, 30–41 min. In both cases, fractions were collected at 1-min intervals at 4°C throughout. The Prot20 column was run using an AKTA-FPLC system with a 280 nm UV absorbance detector (GE-Healthcare). The column was equilibrated with Buffer 1 (20 mM sodium phosphate, pH 7.4, 0.15 M NaCl) for at least two column volumes. Buffer 2 was 0.1 M glycine, pH 2.5, 0.1%-β-d-octylglucopyranoside. For the Prot20 column, 100 μL neat, filtered serum was injected per run with conditions as follows: max. pressure, 0.5 MPa, 100% Buffer 1, 0.3 mL/min, 0–22 mL, 100% Buffer 2, 3.0 mL/min, 22–52 mL, 100% Buffer 1, 3.0 mL/min, 52–82 mL. In all, 1 mL fraction was collected at 4°C throughout.

For each run, fractions containing the depleted serum (peak 1) following removal of the high-abundance proteins were pooled and frozen at −80°C as were fractions containing the bound high-abundance proteins (peak 2). For peak 1, these were typically fractions 4–7 (MARS6, 2 mL), 9–16 (MARS14, 1 mL), and 10–20 (Prot20, 11 mL). For peak 2, these were typically fractions 14–16 (MARS6, 3 mL), 23–25 (MARS14, 3 mL), and 26–43 (Prot20, 18 mL). After each run, the columns were re-equilibrated (Buffer A, 10 min, 1 mL/min for MARS6 and MARS14; Buffer 1, 10 min, 1 mL/min for Prot20). At least one blank run was performed between each serum injection to maximise column reconditioning and minimise carryover. For analysis, peaks 1 and 2 (formed as above) were concentrated using 15 mL capacity, 10 kDa MWCO filters (Sigma-Aldrich) for centrifugation at 4000×*g* for 5–15 min. The concentrated material was then desalted (for MS) or desalted and buffer exchanged into DIGE buffer (7 M urea, 2 M thiourea, 4% CHAPS) using 2 mL 7 kDa MWCO ZEBA spin-desalting columns according to the manufacturer's instructions.

### 2.4 Top-down assessment of reproducibility, specificity, and depth of coverage using 1-D SDS-PAGE and 2-D DIGE

Peak 1 material was combined from four runs of each column to give sufficient protein for downstream gel-based analyses and a similar process was carried out for peak 2 material. Each such pool was considered as one replicate in the experimental design (Fig. 1A).

#### 2.4.1 1-D PAGE

Examination by 1-D PAGE as a gross assessment was initially carried out. Both peak 1 (1 μg protein/gel lane) and peak 2 (5 μg protein/gel lane) from four replicates were subjected to separation by 1-D PAGE in 18×16 cm 12% gels using the SE600X electrophoresis system (Hoeffer, MA, USA) and subsequently silver stained using the ProteoSilver™ Silver Stain Kit (Sigma-Aldrich), according to the manufacturer's instructions.

#### 2.4.2 2-D DIGE

Two separate DIGE experiments were performed, one comparing the peak 1 samples from all the immunodepletions, and one for the peak 2 samples. In each case, an internal standard was used formed from equal amounts of material from each replicate from each column. Four replicates of peak 1 and peak 2 for each of the three columns in DIGE-labelling buffer (7 M urea, 2 M thiourea, and 4% w/v CHAPS) were adjusted to a protein concentration of 1 mg/mL for lysine DIGE labelling with fluorescent dyes Cy2, Cy3, or Cy5 (GE Healthcare). Samples were buffered with 2.5 μL of 1 M Tris-HCl, pH 9.5, per 50 μg of protein to pH 8.5–9.5 estimated with pH-Fix 4.5–10 strips (Fisher Scientific, UK). Diluted Cy-dye (1:4 in anhydrous DMF) was used to label samples in the ratio of 50 μg protein:200 pmol dye for 30 min on ice. An aliquot of 10 mM lysine (1 μl/50 μg labelled protein) was then added and the sample was incubated on ice for 10 min to allow coupling to any unreacted dye. An equal volume of buffer containing 7 M urea, 2 M thiourea, 4% w/v CHAPS, and 1.6% v/v Pharmalyte 3–10, 2% w/v DTT was then added to each sample and the samples were incubated at room temperature for 15 min. Samples were combined with an internal standard consisting of equal amounts of depleted material from each column and an appropriate volume of a buffer containing 7 M urea, 2 M thiourea, 4% w/v CHAPS, 0.8% v/v Pharmalyte 3–10, and 1% w/v DTT was added to make a total volume of 450 μL containing 75 μg labelled protein per dye/gel strip with 10 μL 0.25% w/v bromophenol blue added to colour the samples. IEF was performed on 24 cm pH 3–10 non-linear gel strips using an Ettan IPGphor™ 3 (both from GE Healthcare). Active re-hydration at 30 V for 18 h was followed by the following gradient program: 500 V for 1 h, gradient to 1 kV over 5 h 30 min, gradient to 8 kV over 3 h, then 8 kV for 14 h (total program time, 130 kVh). After focussing, strips were incubated for 15 min with 10 mL/strip of equilibration buffer (6 M urea, 30% v/v glycerol, 2% w/v SDS in 0.05 M Tris-HCl, pH 6.8) supplemented with 10 mg/mL DTT, followed by alkylation with 10 mL equilibration buffer/strip supplemented with 40 mg/mL iodoacetamide (IAA). Second-dimension separation was achieved using 24 cm 10% polyacrylamide gels. Strips were placed on the gels and overlayed with molten 1% w/v low-melting-point agarose and electrophoresis carried out in an Ettan DALT*twelve* Separation Unit (GE Healthcare) tank at 1.5 W/gel overnight with 1× Tris-glycine running buffer (25 mM Tris, pH 8.3, 192 mM glycine, 0.1% w/v SDS), and 2.5× Tris-glycine running buffer above the gels. Gels were then scanned on a Typhoon Trio (GE Healthcare) in fluorescence acquisition mode, using 1000 micron pixel size to test the photomultiplier tube (PMT) voltage settings and 100 micron pixel size for final scans. Focal plane was set to +3 mm, and laser settings were selected to correspond to the dyes being used. PMTs were adjusted to achieve intensity of 80–90 000 in the spot used for normalisation (the second most abundant spot on each gel). DIGE images were analysed using Progenesis SameSpots V3.3 (Nonlinear Dynamics, Newcastle-upon-Tyne, UK), being superceded later with V4 for the alignment of preparative gels and generation of spot-picking lists.

#### 2.4.3 Identification of proteins spots

Preparative gels were generated as above but using 200 μg unlabelled protein for peak 1 material and 150 μg unlabelled protein for peak 2. Gels were as above, but were backed with polyester backing (PAG backing – Lonza, UK) and were silver stained using a modified form of the ProteoSilver™ method (lower percentage alcohol treatments) to minimise gel volume alterations and avoid detaching of the gels from the PAG backing. Picking list files were imported from Progenesis SameSpots into the Ettan Spot Picker V1.2 software and gel spots (diameter, 1.4 mm) were obtained directly using the robotic spot picker into in a 96-well plate in water. Gel pieces were equilibrated in 100 mM ammonium bicarbonate and then reduced/alkylated with DTT/IAA, respectively, followed by incubation with 40 ng/μL sequencing grade-modified trypsin (activity 20 388 U/mg, Promega) overnight. Supernatant from the overnight digestion was collected into fresh tubes and the gel pieces were extracted with water for 10 min in a sonicating water bath, followed by a repeat extraction for 10 min with 50% v/v ACN/1% v/v formic acid. Extraction was performed twice in total and all the extracts were combined. The peptide extract was frozen (−20°C), dried using a Speedvac, and the samples were stored at −80°C until analysis. Tryptic digests were analysed by LC-MS/MS using a nano-HPLC (Agilent, USA) QSTAR-XL quadrupole time-of-flight hybrid mass spectrometer (Applied Biosystems, UK) as described previously [15], except that the LC/MS/MS raw data were processed by Analyst v2.0 and a script plug-in Mascot.dll 1.6b24 (Applied Biosystems), and the MS/MS spectra were sent to the local MASCOT database search engine (v2.2, Matrix Science, UK) for database searching with the following parameters: IPI human protein database (version 3.48, 71 401 sequences); precursor mass tolerance, 0.15 Da; fragment ion mass tolerance, 0.1 Da; enzyme, trypsin/P; max missed cleavage site, 1; Peptides with scores above identity level (*p*<0.05) were considered identified. Protein identifications required at least one unique significant peptide (*p*<0.05) and were filtered to remove keratins and trypsin autolysis peptides.

### 2.5 Bottom-up assessment of protein removal and depth of coverage using LTQ Orbitrap Velos LC-MS/MS analysis

For the initial LC-MS/MS studies, each technical replicate (prepared from pooled peak 1 material from four runs of each column) or whole serum was analysed (three injections per replicate) by LC-MS/MS following processing using the Filter Aided Sample Preparation (FASP) method [16] as follows. All centrifugation steps were performed at 14 000×*g*. In total, 250 μg of each sample (132 μg in the case of Prot20 column) was heated at 95°C with 50 mM DTT, centrifuged for 5 min, and applied to a 30-kDa MWCO filter (Amicon Ultra-0.5 Centrifugal Filter Unit with Ultracel-30 membrane, Millipore). The sample was washed on the filter with 250 μL of 50 mM ammonium bicarbonate. Briefly, 80 μL of 120 mM IAA was added to the filter and the samples were incubated in the dark for 10 min. After a 5-min centrifugation step, the sample was washed on the filter four times with 250 μL of 50 mM ammonium bicarbonate. Porcine trypsin (activity 20 388 U/mg, Promega) was re-suspended in 40 μL 50 mM ammonium bicarbonate and 8 μL of this solution was added to each sample. After overnight incubation with a fresh collection tube at 37°C, filters were washed with 40 μL, then 100 μL, water, and the filtrate was collected by centrifugation.

The samples were analysed twice, first using a 4-h LC gradient and then repeated using an extended 5-h gradient (MARS14- and Prot20-depleted samples only) with three injections for each sample in each case. For the first runs, the peptide mixture was separated by nanoscale C18 reversed-phase LC (Easy-nLC, ThermoScientific, Bremen, Germany) coupled on-line to an LTQ-Orbitrap Velos mass spectrometer (ThermoScientific). An aliquot of 1 μg material was loaded onto a peptide captrap (Michrom Bioresources, Auburn, CA, USA) using water containing 0.1% formic acid (mobile phase A) at 10 μL/min. Peptide separation was performed on a pulled tip column (15 cm×100 μm id) containing C18 reprosil 5 μm particles (Nikkyo Technos, Tokyo, Japan) using increasing amount of ACN containing 0.1% formic acid (mobile phase B) at 300 nL/min. Gradient conditions were as follows: 5–34% B (0–210 min), 34–50% B (5 min), 50–80% B (3 min), 80% B held for 4 min, 80–5% B (3 min), and the total run time was 240 min. The mass spectrometer was operated in positive ion mode and a data-dependent ‘Top 20’ method was employed. In each cycle, a full-scan spectrum was acquired in the Orbitrap at a target value of 1E6 ions (2 microscans) with resolution *R*=30 000 at *m*/*z* 400 followed by ion-trap CID on the 20 most intense ions with a target value of 5E3 ions (1 microscan). The ‘lock mass’ function was enabled for the MS mode, where the background ion at *m*/*z* 445.1200 was used as the lock mass ion. General MS conditions were as follows: spray voltage, 1.75 kV; no sheath or auxiliary gas flow; S-lens, 60%. FT preview mode was disabled, charge-state screening enabled, and rejection of singly charged ions enabled. Ion selection thresholds were 5000 counts for MS2, 35% normalised collision energy, activation *q*=0.25, and activation time of 10 ms were applied for CID. Dynamic exclusion was employed and ±5 ppm window of the selected *m*/*z* was excluded for 20 s. Samples were analysed in triplicate, and the results were combined. The MASCOT program version 2.2.04 was used to generate up to ten peptide sequence candidates per fragmentation spectrum (Matrix Science), and International Protein Index (IPI) version 3.48 was searched. For database searching, maximum peptide mass deviation was 7 ppm and *m*/*z* 0.5 units for fragmentation peaks (optimal for linear ion-trap data). Data analysis was performed using the MaxQuant software (version 1.0.13.13) [17]. Filtering was done at 1% FDR at the peptide level, and 5% FDR at the protein level. Carbamidomethylation was set as a fixed modification, with protein N-terminal acetylation and oxidation of methionine as variable modifications, enzyme: trypsin/P, maximum number of missed cleavages: 2. Ingenuity Pathways Analysis (IPA) software was used subsequently to analyse the location annotations for the protein lists produced. Where multiple UniProt numbers were associated with an entry on the IPI human 3.48 database, one entry only was used for IPA analysis to avoid skewing data using multiple accession numbers for non-unique peptides.

For the longer LC separation (5 h), the peptide mixtures were acidified to the final 0.1% TFA concentration and 1 μg of peptides per each run was used. The LC-MS/MS analytical setup was similar to that described previously [18]. The LC setup was connected to LTQ Orbitrap Velos mass spectrometer equipped with a Proxeon nanoelectrospray ion source. The samples were injected directly onto an in house 25 cm capillary emitter column (75 μm id, packed with 3.5 μm Kromasil C18 media), using Dionex UltiMate 3000 RSLCnano system at a flow rate of 0.5 μL/min. The total acquisition time was 300 min, the major part of the gradient (from 10 to 270 min) being 3–25% ACN in 0.1% formic acid at a flow rate of 0.4 μL/min. Data-dependent acquisition was implemented. Survey MS scans were acquired in the Orbitrap with the resolution set to 60 000. Up to 30 most intense ions per scan were fragmented and analysed in the linear trap. The data analysis was again performed using the MaxQuant software [17].

### 2.6 Bottom-up assessment of reproducibility using LTQ Orbitrap Velos LC-MS/MS analysis

Based on the results found, the MARS14 and Prot20 columns were then selected for the examination of reproducibility by LC-MS/MS. This was carried out using a similar pooled serum sample (protein concentration, 48.7 mg/mL) with three independent (different days) technical replicates of the column depletion in each case followed by analysis by LC-MS/MS as above using three injections but with 4-h LC run for each injection (Fig. 1B). Additionally, to be able to examine the reproducibility of the LC-MS/MS per se, analysis of one replicate from each of the columns was repeated on three separate occasions (three separate analyses, three injections each time). The label-free quantitation algorithm within the MaxQuant software (version 1.1.1.21) was used.

## 3 Results

### 3.1 Recovery and initial reproducibility assessment

The amount of protein recovered in peak 1 decreased with increasing immunodepletion of the serum pool (starting protein concentration of 67.3 μg/μL) as expected with mean percentage recoveries of 11.2, 7.8, and 4.5% for MARS6, MARS14, and Prot20, respectively for 24, 24, and 16 runs and corresponding CVs of 6.0% for MARS6, 10% for MARS14 but increasing to 16% for Prot20. Two blank runs were necessary to minimise carry-over for the MARS6 column, and one for the MARS14 and Prot20 columns.

Reproducibility of the immunodepletion procedure at a gross level was assessed by examination of the UV absorption traces and 1-D SDS-PAGE (Supporting Information Figs. 1 and 2) which demonstrated good reproducibility. Reproducibility was also assessed using 2-D DIGE for peaks 1 and 2 by examining the CVs of spot-normalised volumes for each peak and each column (24 gels i.e. four replicates in each case; Supporting Information Fig. 3). Distributions of CVs for protein spot intensities with the peak 1 and peak 2 2-D DIGE gels were all positively skewed. Median CVs were similar across all three columns at 26.4% (MARS6), 28.4% (MARS14), 25.3% (Prot20) for depleted (peak 1) material, and 28.5% (MARS6), 36.2% (MARS14), 31.1% (Prot20) for bound (peak 2) material.

### 3.2 2-D DIGE analysis of the depleted material

Distinct gel profiles were seen for each column type (Supporting Information Fig. 4). Standard DIGE experimental analysis was performed within the Progenesis SameSpots software, including selection of reference image, alignment, and editing. Column specific sub-experiments were also created where each spot could be assessed for inclusion looking only at that column class (MARS6, MARS14, or Prot20), using aligned DIGE images from the main experimental analyses, to facilitate spot counting. For the depleted material (peak 1), this gave 1186 spots (MARS6), 1137 spots (MARS14), and 1149 (Prot20). For the bound material (peak 2), this gave 750 spots (MARS6), 919 spots (MARS14), and 969 spots (Prot20).

PCA using only those spots with significant differences between groups by ANOVA with *p*<0.05 showed good separation between columns for both peak 1 and peak 2 experiments, indicating that the profile continues to alter with increasing immunodepletion as expected (Supporting Information Fig. 4). The first two principal components explained approximately 75% of the total variation in the data.

A spot classification dendrogram was used to identify peak 1 spots which were significantly different between (i) MARS6 and all other groups; (ii) MARS14 and all other groups; (iii) Prot20 and all other groups; (iv) MARS6 and MARS14; (v) MARS14 and Prot20; (vi) MARS6 and Prot20; and (vii) spots displaying stepwise increase with increasing immunodepletion. A cut-off of ≥4-fold change in the direction of increasing immunodepletion was used to create lists of spots of interest from these groups resulting in a non-redundant list of 123 spots. Following manual quality control assessment, 76 spots were cut from preparatory gels for sequencing (Supporting Information Fig. 5a). However, none of these was found to be of significantly lower abundance or major potential interest with many representing additional isoforms of previously visualised proteins (Supporting Information Data 1, Supporting Information Data 4, and single peptide spectra are available at http://www.proteomics.leeds.ac.uk/supplementary_data/immunodepletion).

### 3.3 Specificity of the immunodepletion

Specificity was assessed on the basis of DIGE profiles of peak 2 proteins essentially as described above for peak 1. Non-redundant lists of proteins appearing sequentially with each depletion were produced but given the number of spots it was not possible to sequence all proteins. Publicly available 2-D PAGE maps of human serum and plasma were used to potentially identify the locations of those proteins expected to be present based on the columns used and these spots were also added to this list for potential-sequencing confirmation. A total of 87 protein spots were selected for sequencing following quality control (Supporting Information Fig. 5b). Identifications were consistent with the respective column depletions in all cases (Supporting Information Data 4, and single peptide spectra are available at http://www.proteomics.leeds.ac.uk/supplementary_data/immunodepletion).

### 3.4 Efficiency of high-abundance protein removal by the immunodepletion columns

In the peak 1 DIGE gels, among the spots cut in analysing significant changes, tryptic peptides compatible with immunoglobulin light and heavy chains, albumin, α_1_-antitrypsin (all columns), and α_2_-macroglobulin and α_1_-acid glycoprotein-1 (MARS14 and Prot20) were observed, indicating incomplete removal. LC-MS/MS analysis of peak 1 material also revealed evidence for the presence of all the depleted proteins at the ≥2 peptide/protein level within the peak 1 samples with the exception of transferrin for the MARS6 column (not seen at even the single peptide level) and transthyretin for the MARS14 column which were not detected. It was very evident, however, that even when proteins which were nominally depleted were detected, the number of peptides found was much reduced compared with those found in the whole-serum sample (Supporting Information Data 2 and 3).

### 3.5 Depth of serum proteomic coverage within peak 1 samples assessed by LTQ Orbitrap Velos LC-MS/MS

**Figure 2:**
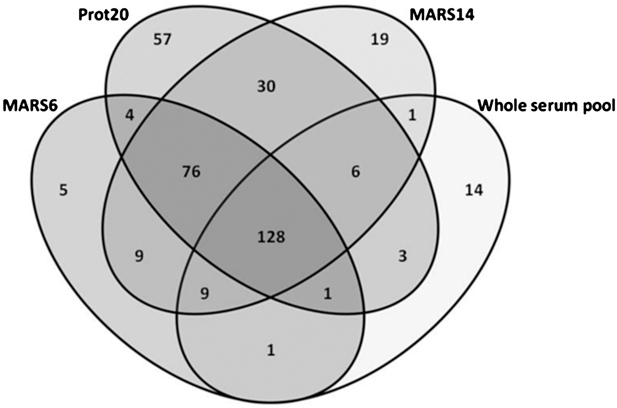
Venn diagram of protein identifications from the 1-D LC-MS/MS LTQ-Orbitrap analysis at ≥2 unique peptides.

The total number of protein identifications found within each immunodepleted sample increased with each increasing degree of immunodepletion (Table 1), for example from 159 (266) in whole serum to 301 (442) with Prot20 at the ≥2 (≥1) unique peptide levels, respectively. Full details of the protein/peptide identifications observed are provided in Supporting Information Data 5. Considerable overlap was observed between intact serum and the three different immunodepleted samples in terms of the proteins observed (Fig. 2); however, both the MARS14 and PROT20 peak 1 samples contained substantial numbers of identities found only in those samples – 19 proteins in MARS 14 peak 1, and 57 proteins in Prot20 peak 1 at the level of two significant peptides (this increased to 9 (MARS6), 25 (MARS14), and 60 (Prot20) if single peptide results were also included). When the MARS14 and Prot20 columns were further compared in the detailed reproducibility study, similar findings were obtained with a high level of overlap and 35 proteins being found only with the Prot20 column at the ≥2 peptide level (18 at the 1+ level), and 29 proteins were found only with the MARS14 (33 at the 1+ level).

**Table 1:** Protein identifications observed within the immunodepleted peak 1 samples and the whole-serum pool when using the LTQ-Orbitrap Velos at the 1 and ≥2 unique peptide levels, with either 4 or 5 h LC-MS/MS runs, and with three injections per sample

Sample	Identifications (4 h run)			Identifications found only in that sample (2 unique peptides, 4 h run)	Identifications (5 h run)		
			
	1 Unique peptide	≥2 Unique peptides	Total		1 Unique peptide	≥2 Unique peptides	Total
Whole-serum pool	107	159	266	14	N/A	N/A	N/A
MARS6 peak 1	127	234	361	5	N/A	N/A	N/A
MARS14 peak 1	133	272	405	19	126	306	432
Prot20 peak 1	141	301	442	57	115	311	426

**Figure 3:**
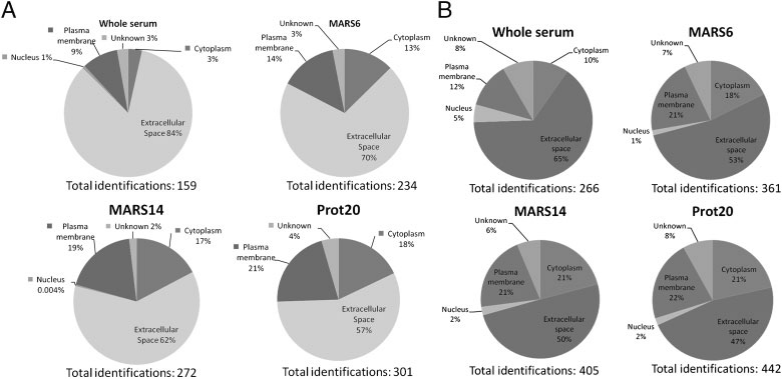
Predicted localisation of proteins identified in the peak 1 immunodepleted material by LTQ Orbitrap Velos analysis. Localisation predictions were obtained from IPA (A) at the ≥2 unique peptide level and (B) at one unique peptide level. Total identifications at the relevant peptide level are given under each pie chart.

Analysis of the origin of the proteins using IPA demonstrated a progressive increase with increasing immunodepletion in the number of proteins in peak 1 with a predicted cytoplasmic/plasma membrane localisation and a concomitant decrease in the number of extracellular proteins (Fig. 3) which was similar for all MS analyses. Many examples illustrate that an increasing degree of immunodepletion coupled with high mass accuracy MS with only a single LC step can begin to enable the detection of moderate to lower abundance proteins (Table 2) such as NGAL, pyruvate kinase M1/M2, carbonic anhydrase III (MARS14 only) interleukin 1 (IL-1) receptor-like protein, carbonic anhydrase I and II, SPARC/osteonectin (MARS14 and Prot20), IL-6 receptor subunit β, macrophage CSF 1 receptor, fatty acid-binding protein 4, vinculin (Prot20 only), and transforming growth factor β receptor (Prot20 only, 1+ peptide level, MS spectrum supplied at http://www.proteomics.leeds.ac.uk/supplementary_data/immunodepletion).

**Table 2:** Selected proteins of moderate–lower abundance from 1-D LC-MS/MS analysis of peak 1 immunodepleted material

Number of proteins depleted	Length of LC-MS/MS run (h)	Protein name	Expected location(s)	Significance/functional comments	Concentration in normal serum where known
20	4	IL-6 receptor subunit β	Membrane/secreted	Signal transduction, immune response	453 pg/mL [34]
		Hypoxia up-regulated protein 1	Endoplasmic reticulum	Cytoprotective	–
		Macrophage CSF 1 receptor	Membrane/secreted	Transmembrane receptor for CSF-1 and IL-34	–
		IL-1 receptor	Membrane	Links to NF-κB pathway and immune response	–
		Fatty acid binding protein 4	Cytoplasm/nucleus	Biomarker of acute kidney injury	13.3–18.3 μg/L [35]
		Vinculin	Cytoplasm/cytoskeleton	Cell adhesion	–
		Transforming growth factor β receptor	Membrane	TGF-β receptor, immune response	0.1–21.0 ng/mL [36]
14	4	Pyruvate kinase M1/M2	Cytoplasm/ nucleus	Known tumour biomarker. Involved in apoptosis	M2 7.2 U/mL [37]
		Protein S A100 A8	Cytoplasm/cytoskeleton/membrane/secreted	Calcium binding, antimicrobial, pro-inflammatory, up-regulates IL-8 and cell surface ICAM1	A8/A9 complex 0.77 μg/mL [38]
		CD59 glycoprotein	Membrane	Inhibition of membrane attack complex, signal transduction for T-cell activation	–
		Transferrin receptor protein 1	Membrane/secreted	Cellular uptake of iron	–
		Profilin-1	Cytoplasm/cytoskeleton	Binds actin and affects cytoskeletal structure	–
	5	Osteopontin	Secreted	Type I immunity	309–413 pg/mL [39]
		Inducible T-cell co-stimulator ligand (B7-related protein 1)	Membrane	Co-stimulator for T-cell proliferation/cytokine secretion	–
		IL-1 receptor accessory protein	Membrane/secreted	Cytokine signalling	>300 ng/mL
14 and 20	4	Neutrophil gelatinase-associated lipocalin	Secreted	Known biomarker of renal injury	76.3 ng/mL [40]
		Secreted protein acidic and rich in cysteine (SPARC)	Basement membrane/secreted	Possible regulator of cell growth	66.8 ng/mL [41]
		Intercellular adhesion molecule 2	Membrane	Adhesive interactions important for antigen-specific immune response	400 ng/mL [42]
		Macrophage mannose receptor 1	Membrane	Involved in phagocytosis by macrophages	–
		Insulin-like growth factor (IGF) binding protein 7	Secreted	Growth factor	–
		Hepatocyte growth factor-like protein	Secreted	Cell proliferation and differentiation, macrophage activation	–
6, 14 and 20	4	Neutrophil defensins 1–3	Secreted	Defensins 1 and 2 have antimicrobial activities	42 ng/mL – for defensins 1–3 combined [43]
		CD166 antigen	Membrane	Expressed by activated T and B cells	–
		Matrix metalloproteinase 9	Secreted	Local proteolysis of ECM, leucocyte migration	16.15 ng/mL [44]
		E-cadherin	Cell junction/membrane	Regulating cell adhesion, mobility/proliferation of epithelial cells	Soluble plasma form – median 3.53 ng/mL [45]

Previously reported concentrations in normal serum are shown where known to illustrate likely abundance range while recognising that concentrations may be increased in the samples analysed here. All were present at ≥2 unique peptides with the exception of TGF-β receptor which was a single peptide.

The extended (5 h LC) analysis of depleted MARS14 and Prot20 samples showed considerable overlap – approximately 92% of the identities for the MARS14 sample were found in the second analysis, and 87% for the Prot20 sample with further identifications including osteopontin, cystatin-M (MARS14 only), and mast/stem cell growth factor receptor (Prot20 only). In the MARS14 peak 1 sample, proteins found previously in the 20 depleted samples with 4-h LC run were also found with MARS14 depletion such as inducible T-cell co-stimulator ligand, IL-1 receptor accessory protein, cell adhesion molecule 1, and lysozyme C (Supporting Information Data 5).

### 3.6 Detailed reproducibility assessment (MARS14, Prot 20, and 1-D LC-MS/MS)

**Figure 4:**
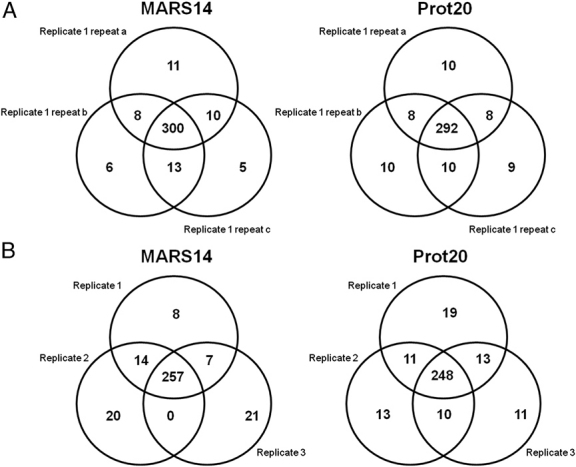
Venn diagrams of protein identifications from the detailed assessment of reproducibility, where each identity is based on ≥2 unique peptides. (A) In each case (MARS14 and Prot20), a depleted sample has been analysed three times through 1-D LC-MS/MS on the LTQ Orbitrap Velos (three injections per each analysis run); (B) In each case (MARS14 and Prot20), a sample has been depleted in three separate replicates of each column, each of which has been analysed by 1-D LC-MS/MS on the LTQ Orbitrap Velos (three injections per run).

**Figure 5:**
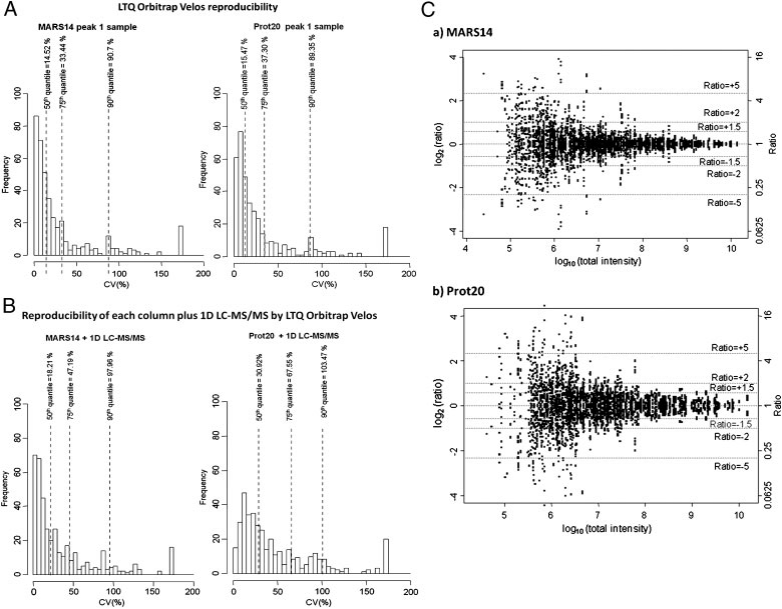
(A) 1-D LC-MS/MS (LTQ Orbitrap Velos) reproducibility – histograms of the CVs of normalised intensities for observed proteins in three repeated analyses (three injections per run) for one depleted peak 1 MARS14 sample and one depleted peak 1 Prot20 sample; (B) Reproducibility of each column plus 1-D LC-MS/MS process as a whole – histograms of the CVs of normalised intensities for observed proteins identified through the whole immunodepletion column plus 1-D LC-MS/MS process (for each of MARS14 and Prot20) in three technical replicates of the whole process; (C) Scatter plots of log_10_(intensity) versus log_2_(ratio) for proteins in every possible pair of analyses within the repeated LC-MS/MS analysis for peak 1 samples from MARS14 and Prot20 columns. Dotted horizontal lines represent different ratios as labelled (+/−).

Reproducibility of the LC-MS/MS aspect examined by repeat analysis of a single sample three times (three injections each repeat) was excellent in terms of proteins identified with 300 and 292 proteins being present in all repeats from a total of 353 and 347 for MARS14 and Prot20, respectively (Fig. 4A). The number of proteins found between replicate immunodepletion column + 1-D LC-MS/MS runs was also remarkably consistent with mean (SD) of 292 (3) and 290 (5), respectively, at the ≥2 unique peptide level and a high degree of overlap in terms of protein identities (Fig. 4B). Similarly, in terms of quantification, the examination of the distribution of %CVs for the normalised intensity for each observed protein showed similar skewed plots as expected for both column samples with 50th, 75th, and 90th quantiles of 14.52, 33.44, and 90.7%, respectively, for the MARS14 peak 1 sample, for example (Fig. 5A). Examination of the reproducibility of each immunodepletion column superimposed on the LC-LTQ Orbitrap Velos process showed only minor to moderate increases in the %CV at each quantile (Fig. 5B), for example 18.21, 47.19, and 97.96% as a comparison to the figures above, showing the relatively high level of reproducibility of the depletion process. However, the 50th, 75th, and 90th quantiles are clearly lower for the MARS14 LC-MS/MS compared with the Prot20 (e.g. 50th quantile 18.21% for MARS14 versus 30.92% for Prot20). Full details are provided in Supporting Information Data 5.

The data from the reproducibility experiment were also analysed in terms of determining appropriate thresholds for future comparative experiments, which could then be used to identify potential biological differences, i.e. changes outside the limit of the technical variability of the process. By plotting the ratio of observed protein intensities against the total intensity in every possible pair of runs (and the reversed pairs for symmetry), scatter plots of the technical variation in ratios (fold changes) can be created (Fig. 5C). While the overall shape of the distributions is the same for both columns, the required ratio as a threshold for the detection of a biological difference would be greater for the Prot20+1-D LC-MS/MS process than for the MARS14+1-D LC-MS/MS process. This is particularly notable at low total intensities.

## 4 Discussion

Immunodepletion is an attractive fractionation strategy for clinical fluid-based biomarker discovery pipelines and in this systematic approach we demonstrate that as a relatively simple and reproducible first step prior to single-dimension LC-MS/MS with the LTQ Oribtrap Velos, low- to moderate-abundance proteins can be found including many proteins of potential biological relevance in this specific disease situation. Such a strategy is compatible with biomarker studies comparing multiple samples in a relatively high-throughput comparison. However, immunodepletion coupled with 2-D DIGE as the downstream analysis is not as beneficial. Distinct profiles with good separation on PCA are readily apparent, but far fewer proteins of interest were revealed with increasing immunodepletion and although additional proteins are found, many represent additional isoforms of more abundant proteins found without immunodepletion. While immunodepletion of eight abundant serum proteins combined with anion-exchange and size-exclusion chromatography has resulted in the detection of some lower abundance molecules (e.g. cathepsins) using 2-D PAGE [19], other 2-D gel-based studies have reported relatively small improvements in being able to visualise lower abundance proteins after immunodepletion [20]. For example, in a study using 2-D DIGE analysis of sera from normal controls and patients with lung cancer following depletion of the 6 most abundant proteins 14 proteins were observed to alter in a statistically significant manner; however, the majority of the proteins identified were forms of haptoglobin or apolipoproteins [21]. This was also found to be the case when immunodepleted samples were subjected to more extensive fractionation based on solution-phase IEF fractionation and narrow range IPG strips prior to 2-D PAGE [22].

Importantly, acceptable reproducibility was observed for all the columns as assessed at a gross level by HPLC/FPLC absorption traces, 1-D gel appearance, and protein recoveries, but also by examining CVs of spot normalised volumes on 2-D DIGE gels. Other investigators have reached similar conclusions for various immunodepletion columns based on such assessments [6, 8, 23, 24] and using other methodologies e.g. SELDI-TOF-MS [8]. Both MARS14 and Prot20 performed reproducibly in terms of the overlap between protein identities observed and also in a detailed analysis of the technical quantitative variability using label-free analysis where it was apparent that the MARS14 column demonstrated less variability than the Prot20 and contributed only a low to moderate increase to the variability of the LC-MS/MS process itself. It was also very apparent that the differences in precision depended on the abundances of the proteins detected, which should be an important consideration when designing and interpreting comparative studies. Similarly, a comparison of MARS6 columns with eight other methods of serum fractionation including size fractionation and Protein A/G separation previously found the MARS6 immunodepletion to be the most reproducible with median CVs of feature intensity values of approximately 11% [6], which at least in part was related to the more automated process with this column compared with the other approaches.

Specificity of depletion as assessed by 2-D DIGE was good as although >700 spots were found in peak 2 fractions for each immunodepletion, many are multiple forms of the targeted proteins. While all proteins sequenced in our study were compatible with expected depletions, this may not be the case for all spots observed as this was a cross-sectional sample rather than a reference map. Concomitant removal of unintended proteins using a MARS6 column has been reported by others [11], and using more sensitive 1- or 2-D LC-MS/MS analyses, multiple non-target proteins have been found to bind to several immunodepletion columns [10, 24–27]. This may be direct non-specific binding, but alternatively it has been demonstrated that multiple peptides/proteins bind to albumin, for example prostate-specific antigen, glycosylasparaginase, and ryanodine receptor 2 [28]. Conversely, by 2-D DIGE, proteins intended to be depleted appeared to be absent from inspection of the gels, but on the analysis by LC-MS/MS, depletion, although very high, was not absolute. Similar results were observed in other LC-MS/MS studies using immunodepletion columns [7, 10, 13, 24–27] and also using ELISA [23] where depletion efficiency was found to be >99%, even with some deterioration of IgG and IgA depletion over time. Similar protein-specific differences in efficiency have been reported for the MARS14 column by LC-MS/MS with α_1_-acid glycoprotein, α_2_-macroglobulin, complement C3, and apolipoproteins being the least efficient [10] although we found C3 and α_1_-acid glycoprotein to be removed more efficiently. What is very important, however, is the consistency of the specific and non-specific protein removal/depletion [10, 25, 26] enabling such approaches to be used in a fractionation scheme.

Although specificity is important and reproducibility even more so, improvements in numbers of proteins identified and depth of coverage is critical. The numbers of serum proteins detected in the current study, given that it is only based on a single-dimensional LC-MS/MS approach, are impressive. This can be contrasted with the lower numbers previously reported for the analysis of serum samples using much more time-consuming extensive and expensive LC/LC-based peptide separation prior to techniques such as iTRAQ, for example. iTRAQ-based comparison of serum samples from normal healthy controls, patients with pancreatic cancer, chronic pancreatitis, and biliary obstruction following removal of the 20 most abundant serum proteins resulted in the identification of 217 proteins [29]. Similarly, immunodepletion of serum from ovarian cancer patients with either the IgY12 HPLC column, the IgY12 spin column, or the MARS6 column combined with LC/LC-MS/MS iTRAQ analysis identified 220 different proteins with a 95% confidence level across the three different immunodepletion strategies [30]. Other studies examining single samples with extensive fractionation through multidimensional LC-MS/MS have reported much greater numbers but not in quantitative comparative studies [25, 27] and even then we have detected some of the lower abundance proteins they describe.

Importantly, we detected a clear trend in increasing protein identifications and increased representation of membrane and intracellular proteins, with increasing immunodepletion. Furthermore, the identifications found with increasing immunodepletion demonstrated proof of principle in that low to moderate concentration and known renal biomarker molecules were detected, e.g. FABP4 and NGAL which is of major current interest as a renal injury biomarker [31]. Using pathological sample pools rather than samples from normal healthy controls in contrast to many immunodepletion evaluation studies [6, 10, 26–28] has really allowed us to evaluate the potential ability of immunodepletion and LC-MS/MS analysis in detecting some of the low- to moderate-abundance molecules which may only be present in disease. The number and nature of these identifications is in keeping with lower abundance protein detection after immunodepletion [11, 26, 27] but in contrast to the limited improvement found in some other studies [14]. The relationship between likely clinical utility and abundance of proteins is difficult and many existing biomarkers lie in the nanogram per milliliter concentration range. Tu et al. [11] argue that their detection of only 23 low-abundance proteins in the <10 ng/mL range (5–6% of their total protein identifications) means low-abundance proteins are likely to remain undetected following MARS7 or MARS14 depletion. However, consideration should be given to the nature of the identifications and their potential relevance as biomarkers, rather than simply the proportion of the identifications they make up and their study was carried out using samples from healthy donors where many proteins present at low abundance even in disease may not be present. Examples from the proteins found only with the more extensive depletions in our study include a number of plausibly relevant molecules for the clinical context from which the serum pool was derived, e.g. IL-1 receptor accessory protein, TGF-β receptor 2, IL-6 receptor subunit β, and macrophage CSF-1 receptor. The ability of the process used here to detect low-concentration proteins of interest is likely to be partly attributable to the use of the high mass accuracy Orbitrap Velos as the downstream analysis platform where even with LC-MS/MS, depth of coverage is superior to that found with much more extensive and time-consuming multidimensional LC-MS/MS approaches in some studies.

Whilst we have focused largely on ≥2 unique peptide-based identifications, information on the numbers of identities at the one unique peptide level (Table 1), as well as their localisation by IPA analysis (Fig. 3B) is also included. The use of higher mass accuracy MS does increase confidence in such identifications although the issue of the use of single peptide-based identification is under debate [32, 33]. These qualitative data are provided to illustrate the possible depth of coverage, and to allow comparison with other data sets. Clearly, any identification at the one unique peptide level would require further confirmation.

These results support the use of the greater levels of immunodepletion (14 or 20 abundant proteins) in potentially revealing biomarker candidates in serum-based clinical discovery experiments when coupled with relatively high-throughput but high mass accuracy LC-MS/MS. Although significant improvements were found with these columns compared with depleting only six proteins, the difference was less apparent moving from depleting 14 to 20 proteins (though further large increases in depletion may be expected to result in even greater depth of coverage, for example depletion of 60 protein [26]). Under such conditions, acceptable reproducibility can be achieved and detection of tissue- and cell-derived products arising from, for example, renal injury, can be found. Based on ease of use (from both automation and lower final volume yield aspects) and better reproducibility, we are now systematically exploring the potential of depleting 14 proteins in carefully designed comparative studies, addressing specific clinical questions to determine to what level new markers can now be discovered using this approach, before consideration of further fractionation strategies in the future.

## Abbreviations

IAA: iodoacetamide
IL: interleukin
IPA: Ingenuity Pathways Analysis
MARS: Multiple Affinity Removal System
MWCO: molecular weight cut-off
