# Supplementary material for: A systematic analysis of the effects of increasing degrees of serum immunodepletion in terms of depth of coverage and other key aspects in top-down and bottom-up proteomic analyses
Source: Proteomics. 2011 Jun;11(11):2222–35. doi: 10.1002/pmic.201100005 (PMC3262148; doi:10.1002/pmic.201100005)
Supplement: Supplementary file 1 [file pmic0011-2222-SD1.pdf]

PROTEOMICS

Supporting Information
for Proteomics
DOI 10.1002/pmic.201100005

Matthew P. Welberry Smith, Steven L. Wood, Alexandre Zougman,
Jenny T. C. Ho, Jianhe Peng, David Jackson, David A. Cairns,
Andrew J. P. Lewington, Peter J. Selby and Rosamonde E. Banks

# A systematic analysis of the effects of increasing degrees of serum immunodepletion in terms of depth of coverage and other key aspects in top-down and bottom-up proteomic analyses

© 2011 WILEY-VCH Verlag GmbH & Co. KGaA, Weinheim 

**Supplementary Data 1.** Examples of proteins identified from the tryptic digest of significantly different spots in the 2D-DIGE experiment. Proteins quoted were seen in more than one preparatory gel, or no conflicting identities were apparent if seen in a single preparatory gel; multiple identities from one spot are separated by "/"; fragments of immunoglobulins observed are not listed

| Increased in: | Number of spots | Examples of proteins identified |
|---|---|---|
| MARS14 and Prot20 compared to MARS6 | 19 | Hemopexin, Proteoglycan 4, Inositol polyphosphate 5-phosphatase, Neutrophil defensin 1, Complement factor B (Fragment) |
| Prot20 compared to MARS14 and MARS6 | 40 | Proteoglycan 4, Complement factor H, Inositol polyphosphate 5-phosphatase, Neutrophil defensin 1 |
| Prot20 compared to MARS6 | 49 | Hemopexin, CD5 antigen-like protein / vitamin D-binding protein precursor, Proteoglycan 4, Complement factor H, Inositol polyphosphate 5-phosphatase, Neutrophil defensin 1 |
| Prot20 compared to MARS14 | 29 | Hemopexin, Inositol polyphosphate 5-phosphatase |
| Stepwise rise (increasing progressively from MARS6 to MARS14 to Prot20) | 7 | Alpha-1B-glycoprotein / Inter-alpha-trypsin inhibitor heavy chain H4 / Afamin |

**Supplementary Data 2: Details of immunodepleted proteins seen with LTQ-Orbitrap Velos**

**Expected depletions**

**Proteins seen in depleted sample on 1D-LC MS/MS Orbitrap Velos analysis**
**(Y = evidence of the protein seen in the sample ; N= evidence of the protein not seen)**

| Expected depletions | | | ≥2 peptide level | | | 1 peptide level | | |
|---|---|---|---|---|---|---|---|---|
| MARS 6 | MARS 14 | ProteoPrep20 | MARS 6 | MARS 14 | ProteoPrep20 | MARS 6 | MARS 14 | ProteoPrep20 |
| Albumin | Albumin | Albumin | Y | Y | Y | Y | Y | Y |
| Transferrin | Transferrin | Transferrin | N | Y | Y | N | Y | Y |
| Fibrinogen | Fibrinogen | Fibrinogen | Y | Y | Y | Y | Y | Y |
| Alpha-1-antitrypsin | Alpha-1-antitrypsin | Alpha-1-antitrypsin | Y | Y | Y | Y | Y | Y |
| Haptoglobin | Haptoglobin | Haptoglobin | Y | Y | Y | Y | Y | Y |
| X | Alpha-2-macroglobulin | Alpha-2-macroglobulin | - | Y | Y | - | Y | Y |
| X | Alpha-1-acid glycoprotein | Alpha-1-acid glycoprotein | - | Y | N[1] | - | Y | Y |
| X | Apo-A-I | Apo-A-I | - | Y | Y | - | Y | Y |
| X | Apo-A-II | Apo-A-II | - | Y | Y | - | Y | Y |
| X | C3 | C3 | - | Y | Y | - | Y | Y |
| X | Transthyretin | Transthyretin | - | N | N | - | Y | N |
| X | X | Ceruloplasmin | - | - | Y | - | - | Y |
| X | X | Apo-B | - | - | N[2] | - | - | Y |
| X | X | C1q | - | - | N | - | - | Y[3] |
| X | X | C4 | - | - | N | - | - | N |
| X | X | Plasminogen | - | - | N | - | - | Y |
| IgG | IgG | IgG | Y[4] | Y[4] | Y[4] | Y[4] | Y[4] | Y[4] |
| IgA | IgA | IgA | Y[4] | Y[4] | Y[4] | Y[4] | Y[4] | Y[4] |
| X | IgM | IgM | - | Y[4] | Y[4] | - | Y[4] | Y[4] |
| X | X | IgD | - | - | Y[4] | - | - | Y[4] |

**Notes**

1. Alpha-1-acid glycoprotein 1 is seen in the Prot20 depleted sample at the 1+ peptide level, but not at the 2+ peptide level. Alpha-1-acid glycoprotein 2 is seen at the 2+ peptide level in all depletions
2. Refers to ApoB (IPI00894122); peptides from (either) Apo B-100 / ApoB-48 are seen at 2+ peptide level - these don't appear to be depleted
3. Complement C1q subcomponent subunit A is seen at the 1 peptide level. Subunits B and C are not seen even at that level
4. Multiple immunoglobulin fragments remain in all depletions at the 2+ peptide level. Distinguishing which are from which immunoglobulin subfamily is difficult

**Supplementary Data 3: Number of unique peptides seen in each peak 1 sample for the immunodepleted proteins.**
**≤1 peptide detections are shown in red**

| Immunodepleted Proteins[1] | WS | MARS 6 | MARS 14 | Prot 20 |
|---|---|---|---|---|
| Alpha-1-antitrypsin | 31 | 30 | 31 | 7 |
| Fibrinogen alpha chain | 14 | 27 | 25 | 12 |
| Fibrinogen beta chain | 15 | 24 | 16 | 7 |
| Fibrinogen gamma chain | 4 | 13 | 6 | 4 |
| Haptoglobin | 22 | 17 | 14 | 12 |
| Serotransferrin | 93 | **0** | 15 | 8 |
| Serum albumin | 113 | 5 | 28 | 45 |
| Alpha-1-acid glycoprotein 1 (Orosomucoid-1) | 11 | 14 | 3 | **1** |
| Alpha-1-acid glycoprotein 2 (Orosomucoid-2) | 9 | 11 | 4 | 2 |
| Transthyretin(Prealbumin) | 5 | 8 | **1** | **0** |
| Alpha-2-macroglobulin | 55 | 73 | 54 | 56 |
| Apolipoprotein A-I | 31 | 58 | 49 | 41 |
| Apolipoprotein A-II | 13 | 15 | 14 | 10 |
| Apolipoprotein(a) | 14 | 33 | 38 | 13 |
| Complement C3 | 22 | 36 | 2 | 4 |
| Apolipoprotein B-100;Apolipoprotein B-48[2] | 52 | 154 | 174 | 157 |
| APOB protein | 2 | 4 | 4 | **1** |
| Plasminogen | 47 | 70 | 74 | **1** |
| Ceruloplasmin | 7 | 11 | 11 | 2 |
| Complement C1q subcomponent subunit A | 4 | 8 | 9 | **1** |
| Complement C1q subcomponent subunit B | 4 | 7 | 6 | **0** |
| Complement C1q subcomponent subunit C | 4 | 8 | 7 | **0** |
| Complement component 4B | **0** | **1** | **1** | **0** |

*1. These are groupings of all the protein names referring to the same protein or fragments / isoforms thereof*
*2. The number of unique peptides seem for these suggests the immunodepletion column antibodies are not directed against these forms*
