# Supplementary material for: A systematic analysis of the effects of increasing degrees of serum immunodepletion in terms of depth of coverage and other key aspects in top-down and bottom-up proteomic analyses
Source: Proteomics. 2011 Jun;11(11):2222–35. doi: 10.1002/pmic.201100005 (PMC3262148; doi:10.1002/pmic.201100005)

Supplementary Figure 1: Protein elution profiles for (a) MARS6 column; (b) MARS14 column; (c) PROT20 column. In (a) and (b) the blue trace represents the UV absorption at 214nm. In (c) the blue trace is UV absorption at 280nm. Representative traces are shown.

a.

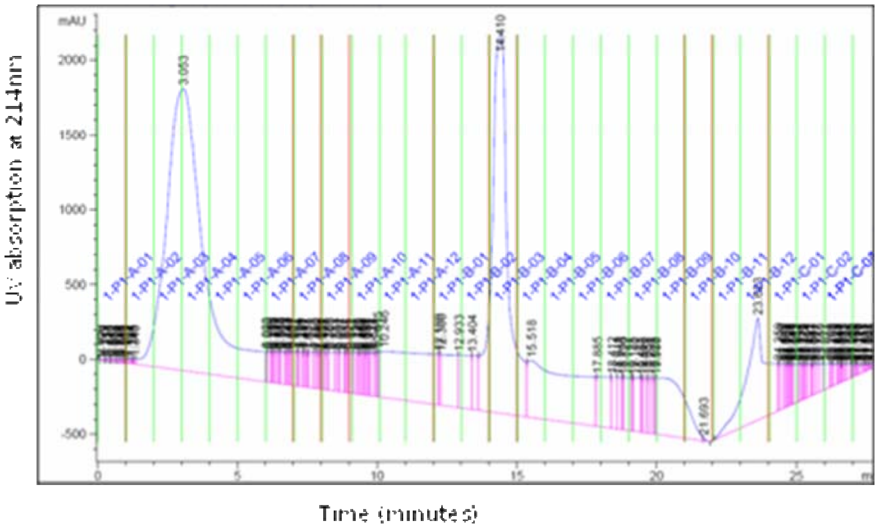

b.

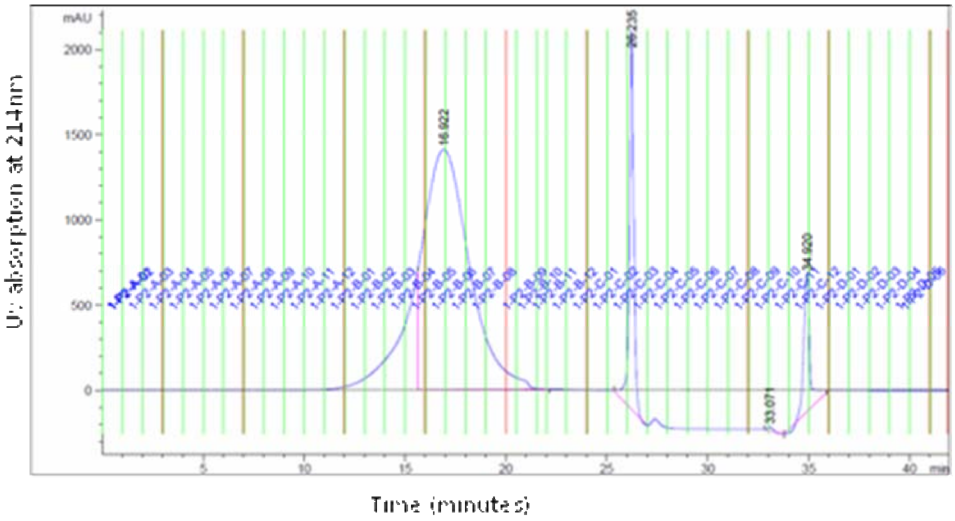

c.

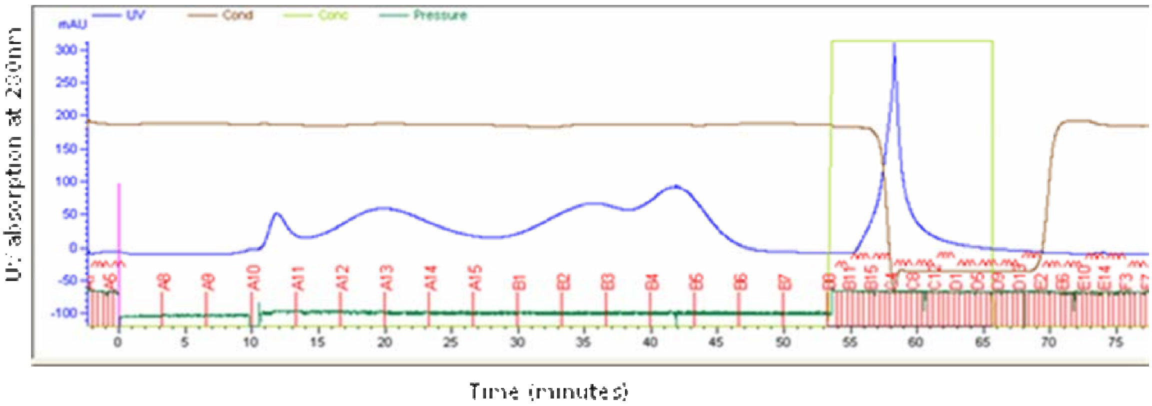

Supplementary Figure 2: Representative examples of 1D-SDS-PAGE analysis showing the reproducibility of immunodepletion of 6, 14 or 20 proteins. Peaks 1 and 2 are shown for each column type, on 12% silver stained SDS-PAGE gels with molecular weight (MW) markers. Peak 1 gels = 1 µg/lane, peak 2 gels = 5 µg/lane.

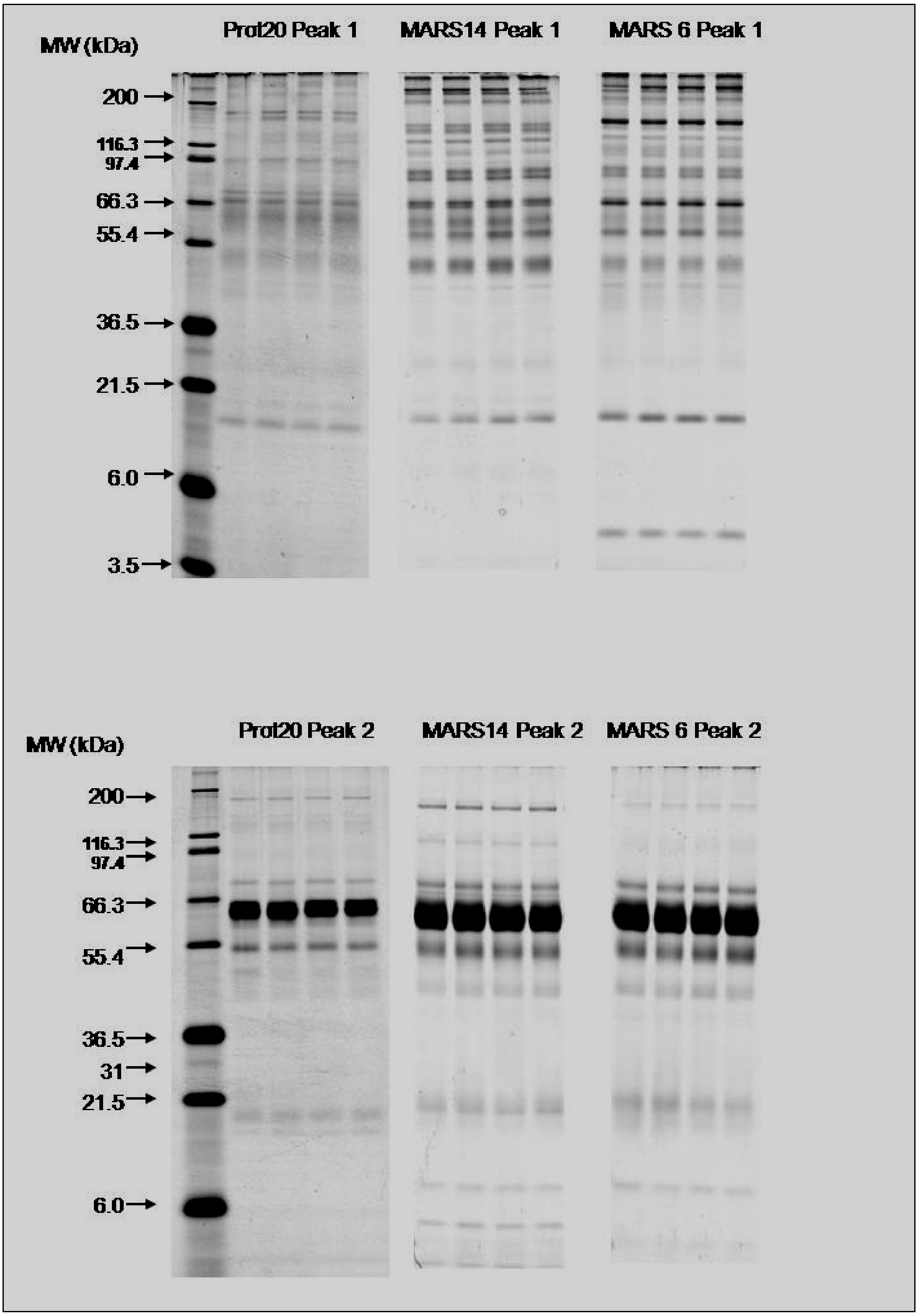

Supplementary Figure 3: Histograms showing distributions of the 2D-DIGE coefficients of variation for gel spot normalised volumes, against scaled frequency measurements (density) derived from 4 replicate gels of peak 1 immunodepleted material per column.

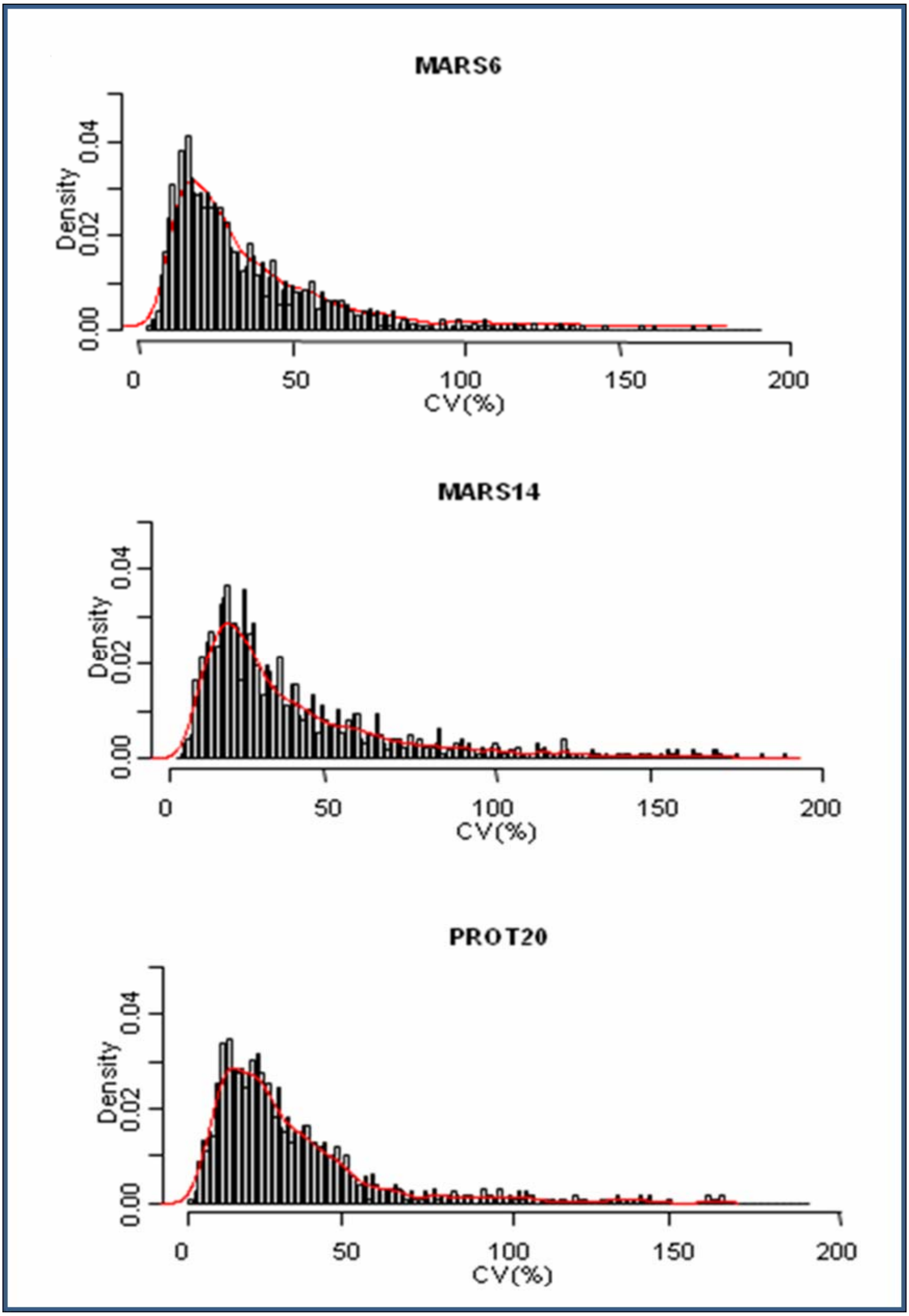

Supplementary Figure 4: Representative 2D gel images for depleted (peak 1; a-d) and bound (peak 2; e-h) material from each column and principal components analysis (PCA) plot for each of MARS6, MARS14, PROT20 columns respectively. On PCA plots, stars mark positions for MARS6 gels, squares for MARS14 and circles for Prot 20. 75μg protein was loaded per DIGE-labelling channel in each case.

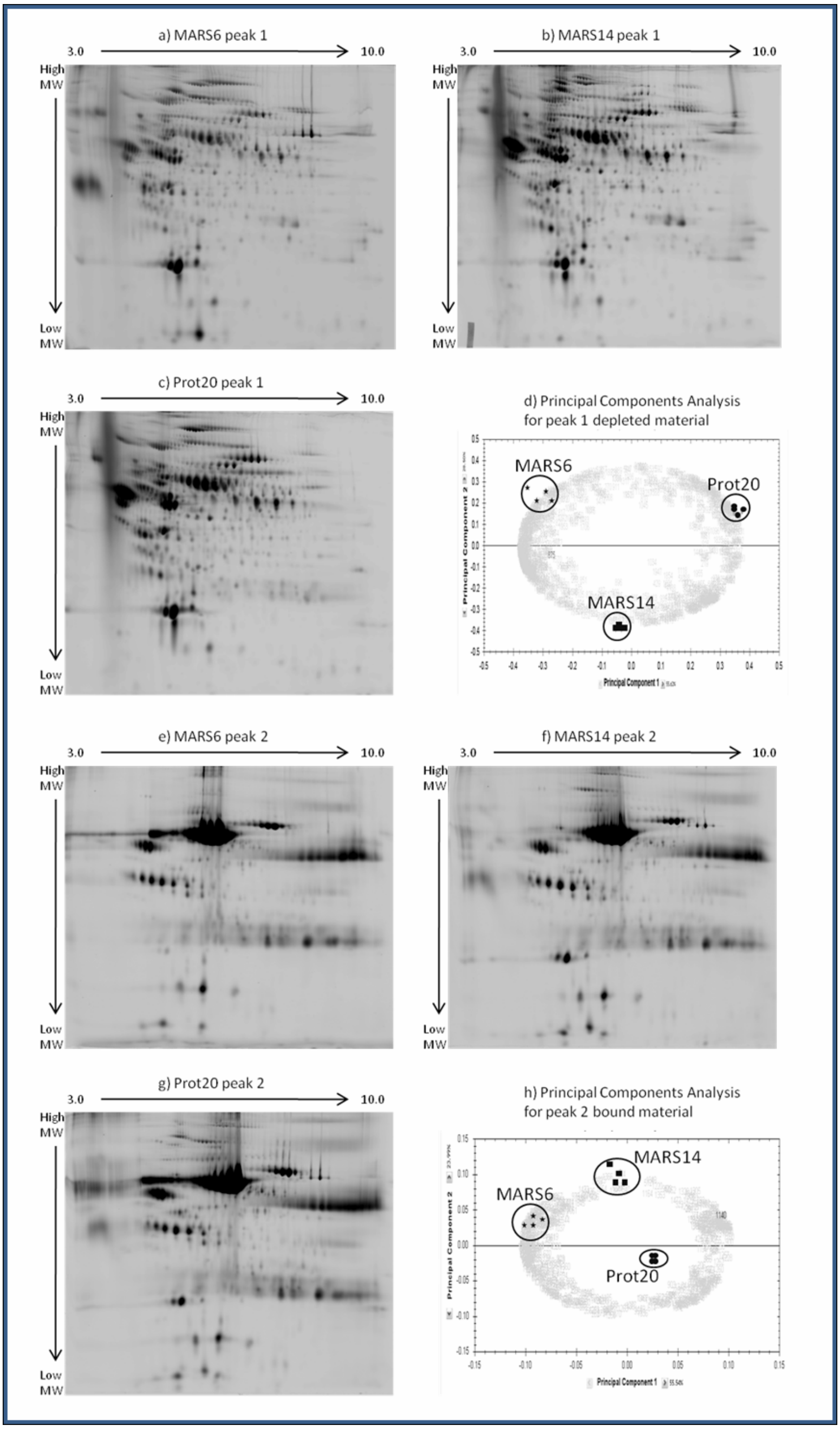

Supplementary Figure 5: a. Peak 1 preparatory gel image with spots selected for cutting marked; **b.** Peak 2 preparatory gel image with spots selected for cutting marked. 150µg prtoein loaded. Both gels silver stained. Spot numbers refer to their designation in the Progenesis SameSpots software used for the DIGE analysis

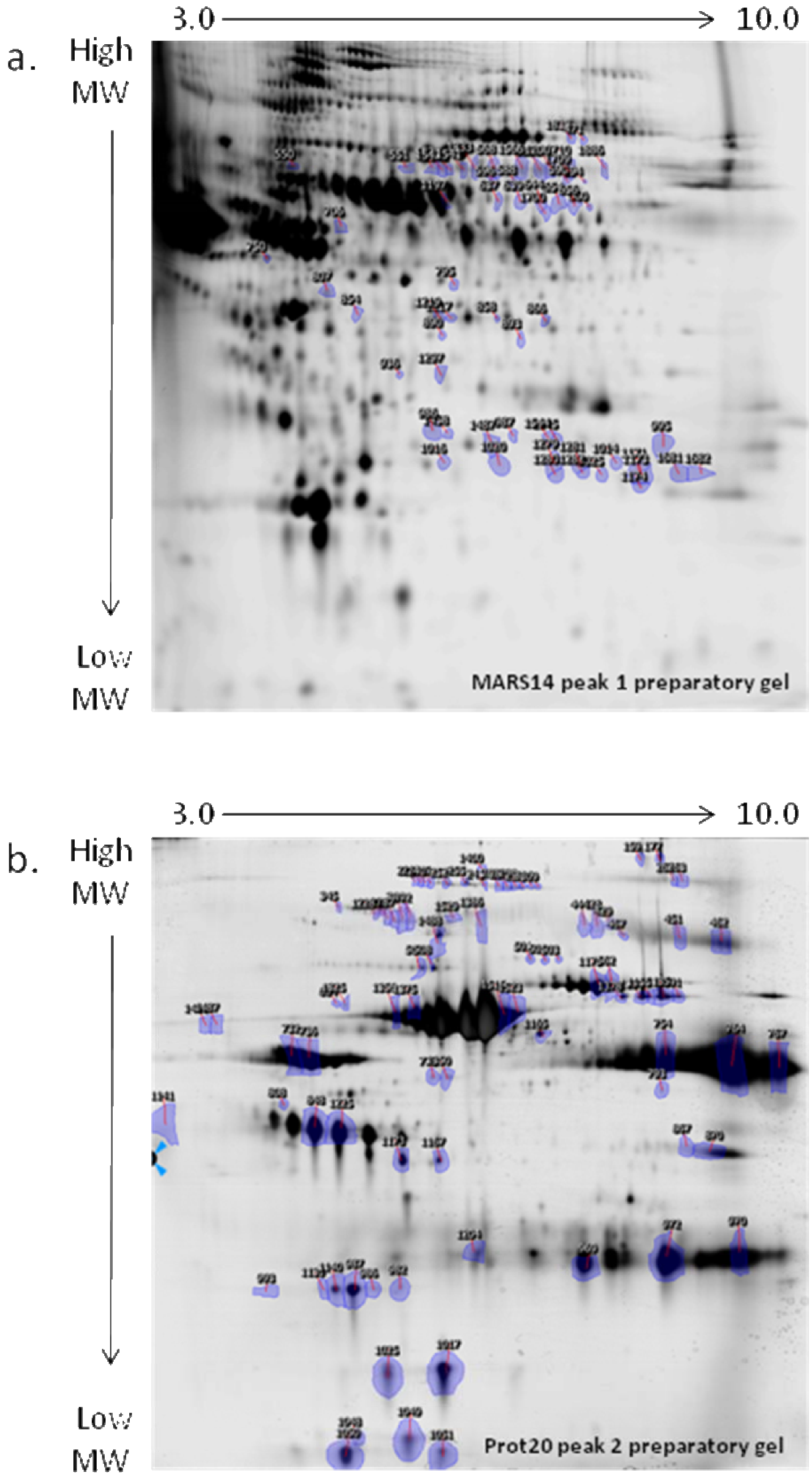

Supplement: Supplementary file 2 [file pmic0011-2222-SD2.pdf]
