# Supplementary material for: A systematic analysis of the effects of increasing degrees of serum immunodepletion in terms of depth of coverage and other key aspects in top-down and bottom-up proteomic analyses
Source: Proteomics. 2011 Jun;11(11):2222–35. doi: 10.1002/pmic.201100005 (PMC3262148; doi:10.1002/pmic.201100005)
Supplement: Supplementary file 3 [file pmic0011-2222-SD3.pdf]

Supplementary Data 4: Peak 1 and 2 MS information for gel spots from preparatory gels. Single peptide spectra are available at www.proteomics.leeds.ac.uk/supplementary_data/immunodepletion

Supplementary Data 5: Protein / peptide identifications from all the LC-MS/MS analyses, i.e. whole serum / MARS6 / MARS14 / Prot20 comparison, MARS14 and Prot20 5h LC gradient runs, and detailed reproducibility for MARS14 and Prot20 (with intensity data)
